# Neuroimaging biomarkers of non-invasive brain stimulation in post-stroke recovery: EEG and fMRI perspectives

**DOI:** 10.1016/j.nicl.2026.104036

**Published:** 2026-07-31

**Authors:** Seoyeon Kim, Sang Wook Lee, Minji Lee

**Affiliations:** a Department of Artificial Intelligence, The Catholic University of Korea, Bucheon, 14662, Republic of Korea; b Department of Biomedical Engineering, The Catholic University of America, Washington, DC, 20064-0001, USA; c Department of Biomedical Software Engineering, The Catholic University of Korea, Bucheon, 14662, Republic of Korea

**Keywords:** Non-invasive brain stimulation, Post-stroke neuroplasticity, Neuroimaging biomarkers, Functional connectivity, Electroencephalography (EEG), Functional magnetic resonance imaging (fMRI)

## Abstract

**Background:**

Non-invasive brain stimulation (NIBS) has emerged as a promising approach to enhance neuroplasticity and functional recovery after stroke. However, substantial inter-individual variability in treatment response limits its clinical translation. The lack of objective neuroimaging biomarkers that capture stimulation-induced brain reorganization remains a critical barrier to personalized neuromodulation.

**Objective:**

To synthesize current evidence on electroencephalography (EEG)- and functional magnetic resonance imaging (fMRI)-based biomarkers of NIBS-induced neuroplasticity in post-stroke populations and to present a unified neuroimaging framework for predicting treatment response and guiding individualized stimulation strategies.

**Methods:**

This review provides a comprehensive and integrative synthesis of studies investigating neuroimaging correlates of NIBS, focusing on EEG and fMRI modalities. Evidence is organized within a unified neuroimaging framework spanning multiple levels of neural organization, including cortical excitability, oscillatory dynamics, and large-scale functional connectivity. Multimodal and computational approaches, such as EEG–fMRI integration and electric field modeling, are also considered to highlight emerging analytical frameworks.

**Results:**

Converging evidence indicates that NIBS induces measurable changes across multiple neural scales. EEG-based metrics, including oscillatory activity, TMS-evoked potentials, and interhemispheric balance indices, reflect modulation of cortical excitability and network dynamics. fMRI findings demonstrate alterations in regional activation and functional connectivity, particularly within motor and association networks. Multimodal approaches further improve the characterization of stimulation-induced reorganization and enhance the prediction of functional recovery.

**Conclusions:**

Neuroimaging biomarkers derived from EEG and fMRI provide critical insight into the mechanisms and variability of NIBS-induced neuroplasticity. Integrating these biomarkers into a unified framework enables stratification of patients and supports the development of precision neuromodulation strategies in stroke rehabilitation. Future research should prioritize large-scale validation and the implementation of biomarker-driven, adaptive stimulation paradigms.

## Highlights

• Neuroimaging biomarkers provide a multi-scale framework for understanding NIBS-induced neuroplasticity after stroke.
• EEG-derived metrics capture cortical excitability and network dynamics associated with functional recovery.
• fMRI measures of functional connectivity and network organization identify systems-level reconfiguration.
• Multimodal integration and computational modeling enable biomarker-driven personalization of neuromodulation.
• A biomarker-guided approach supports the transition toward individualized, circuit-based stroke rehabilitation.

## 1 Introduction

Stroke is increasingly conceptualized not merely as a focal lesion but as a disorder of distributed brain networks, characterized by large-scale disruptions in interhemispheric balance, functional connectivity, and dynamic neural communication (Liu et al., 2024). Although endogenous neuroplastic mechanisms enable partial recovery, many survivors experience persistent motor and cognitive impairments that reflect maladaptive network reorganization rather than isolated cortical damage (Tscherpel et al., 2020, Lee et al., 2022). These systems-level alterations underscore the necessity of therapeutic strategies capable of modulating neural circuits rather than targeting single cortical sites.

Non-invasive brain stimulation (NIBS), including transcranial magnetic stimulation (TMS) and transcranial electrical stimulation (tES), has emerged as a promising neuromodulatory approach for investigating the involvement of targeted brain regions in functional processes and for modulating dysfunctional network organization after stroke (Dayan et al., 2013). However, a fundamental challenge in the clinical application of NIBS is the high inter-individual variability in treatment response. Even among neurologically intact individuals and across standardized plasticity-inducing protocols, motor evoked potentials (MEPs) and cortical excitability responses exhibit significant heterogeneity (Wassermann, 2002, Wiethoff et al., 2014). This variability highlights a limitation of empirically applied stimulation paradigms and emphasizes the need for state-dependent, biomarker-informed intervention strategies that account for individual functional network states and neurophysiological characteristics.

Neuroimaging provides a critical bridge between mechanistic understanding and clinical translation. Electroencephalography (EEG) enables the discovery of oscillatory interaction networks and the characterization of phase-interaction mappings that coordinate distributed neuronal activity (Palva and Palva, 2012). Complementarily, functional magnetic resonance imaging (fMRI), including resting-state and task-based paradigms, delineates large-scale functional brain organization by characterizing regional activation patterns and interactions among anatomically distributed brain regions, thereby providing insights into functional connectivity and network reorganization after stroke (van den Heuvel and Hulshoff Pol, 2010). These modalities offer complementary temporal and spatial perspectives on stimulation-induced neuroplasticity, enabling multi-scale characterization of post-stroke network reorganization. This review specifically focuses on repetitive transcranial magnetic stimulation (rTMS), transcranial direct current stimulation (tDCS), and emerging transcranial alternating current stimulation (tACS) paradigms integrated with EEG and fMRI biomarkers in post-stroke recovery. Other NIBS modalities, such as transcranial random noise stimulation (tRNS), are discussed where relevant but were not systematically reviewed. The present synthesis primarily focuses on scalp EEG methodologies, which remain the dominant electrophysiological approach in post-stroke neuromodulation studies. Future opportunities lie in invasive electrophysiological recordings, which may enable higher-resolution characterization of stimulation-induced mechanisms and circuit-level neuroplasticity.

Building on these advances, this review integrates current EEG and fMRI evidence to establish a neuroimaging-driven framework that links multi-scale biomarkers to neuromodulation mechanisms and clinical translation. Rather than treating EEG and fMRI as independent neuroimaging modalities, this review emphasizes their distinct yet complementary roles in characterizing NIBS-induced neuroplasticity. EEG provides temporally precise measures of cortical excitability, oscillatory dynamics, and rapid electrophysiological responses, whereas fMRI captures spatially resolved changes in regional activity, large-scale connectivity, and systems-level network reorganization. This complementary perspective provides a unified framework for understanding the mechanisms of neuroplasticity, explaining inter-individual variability in treatment response, and guiding biomarker-driven individualized neuromodulation after stroke. Specifically, this review addresses the following key questions:

1. What neurophysiological and network-level mechanisms of NIBS can be characterized by EEG and fMRI biomarkers in post-stroke populations?
2. How do these biomarkers relate to functional reorganization and inter-individual variability in treatment response?
3. How can multimodal neuroimaging and computational approaches be leveraged to guide biomarker-driven personalization of NIBS in stroke rehabilitation?

## 2 Methods

This review was designed as a conceptual and mechanistic narrative review rather than a formal systematic review or meta-analysis. Accordingly, the aim was not to provide an exhaustive quantitative aggregation of all available studies, but to synthesize representative and mechanistically informative evidence linking NIBS, neuroimaging biomarkers, and post-stroke recovery.

A targeted literature identification process was conducted using PubMed and Web of Science to identify studies published between 2015 and 2025. Search terms included combinations of “non-invasive brain stimulation”, “stroke”, “repetitive transcranial magnetic stimulation”, “transcranial direct current stimulation”, “transcranial alternating current stimulation”, “EEG”, “fMRI”, “functional connectivity”, “brain network”, “neuroplasticity”, and “biomarker”. Additional relevant studies were identified from reference lists of key articles when they contributed directly to the conceptual framework of biomarker-guided neuromodulation.

Studies were prioritized for inclusion when they met the following criteria: (1) inclusion of post-stroke human participants; (2) application of NIBS modalities, including rTMS, tDCS, or tACS; (3) use of EEG, fMRI, or multimodal neuroimaging outcomes; and (4) relevance to cortical excitability, oscillatory dynamics, regional BOLD activity, functional connectivity, network topology, dynamic network reconfiguration, or computational modeling. Studies were not selected solely on the basis of intervention efficacy; rather, priority was given to studies that provided mechanistic insight into how NIBS modulates neural activity, connectivity, or network organization after stroke.

Studies were not considered as core evidence when they focused exclusively on non-stroke populations, animal or preclinical models, behavioral outcomes without neurophysiological or neuroimaging measures, or purely technical stimulation methods without relevance to post-stroke recovery mechanisms. Background and methodological references were cited where necessary to define key concepts, but the main synthesis was organized around studies directly informing neuroimaging biomarkers of NIBS effects in stroke.

The selected literature was synthesized according to an a priori conceptual framework rather than a PRISMA-based systematic review structure. Specifically, evidence was organized across multiple analytical levels: local cortical excitability and oscillatory activity, regional BOLD responses, inter-regional functional connectivity, large-scale network topology, dynamic network reconfiguration, and computational or predictive modeling. This framework was used to clarify how EEG, fMRI, and multimodal biomarkers may function as monitoring, predictive, response-related, or safety biomarkers within biomarker-guided stroke neuromodulation.

**Fig. 1:**
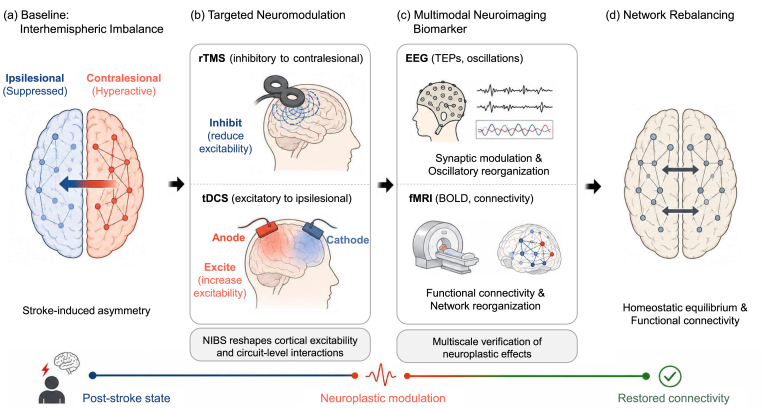
Conceptual framework linking NIBS-induced neuroplasticity to neuroimaging biomarkers in post-stroke recovery. (a) Stroke-induced imbalance characterized by ipsilesional hypoexcitability and contralesional hyperexcitability, reflecting disrupted interhemispheric and network dynamics. (b) Targeted neuromodulation via NIBS protocols reshapes cortical excitability and circuit-level interactions. The illustrated stimulation configurations are representative examples of biomarker-guided neuromodulation strategies and may vary according to patient characteristics and therapeutic objectives. (c) Biomarker-based verification using EEG and fMRI captures complementary electrophysiological and hemodynamic signatures of NIBS-induced neuroplasticity across temporal and spatial scales. (d) Restored network equilibrium and functional recovery, supported by biomarker-validated normalization of large-scale connectivity patterns.

## 3 Mechanistic basis of NIBS-induced neuroplasticity in post-stroke recovery

The therapeutic effects of NIBS in post-stroke rehabilitation arise from its capacity to modulate neuroplasticity across multiple biological scales. At the synaptic level, NIBS induces long-term potentiation (LTP)- and long-term depression (LTD)-like plasticity through activity-dependent mechanisms (Bai et al., 2023a). High-frequency rTMS and anodal tDCS enhance NMDA receptor-mediated signaling, promoting ${\mathrm{Ca}}^{2+}$ influx and strengthening perilesional synaptic efficacy (Ovadia-Caro et al., 2019). These effects are accompanied by upregulation of brain-derived neurotrophic factor (BDNF) and modulation of GABAergic inhibition, collectively lowering the threshold for adaptive cortical reorganization (Liu et al., 2024). As illustrated in Fig. 1, NIBS-induced recovery can be conceptualized as a transition from maladaptive interhemispheric imbalance toward restored network equilibrium, with neuroimaging biomarkers serving as objective indicators of this multi-scale reorganization. This framework aligns with the bimodal balance-recovery model, which proposes that stroke recovery depends on residual structural reserve and interhemispheric interactions (Di Pino et al., 2014).

Beyond local synaptic modulation, NIBS reshapes large-scale network dynamics. Stroke is associated with interhemispheric imbalance and maladaptive connectivity patterns that disrupt coordinated circuit function. Within the interhemispheric inhibition (IHI) framework, excitatory stimulation of the ipsilesional cortex or inhibitory modulation of the contralesional hemisphere seeks to restore transcallosal equilibrium (Hsu et al., 2023). Increasing evidence, however, suggests that NIBS effects extend beyond focal excitability changes to influence distributed motor and cognitive networks, facilitating the recruitment of alternative pathways and mitigating diaschisis (Bajaj et al., 2015).

These multi-level mechanisms provide the biological substrate for measurable neuroimaging signatures. Synaptic potentiation and inhibitory rebalancing manifest as changes in cortical reactivity, oscillatory dynamics, and functional connectivity patterns, forming the basis for EEG- and fMRI-derived biomarkers. Understanding this mechanistic cascade is essential for interpreting neuroimaging readouts and for linking stimulation-induced plasticity to individualized recovery trajectories (Yuan et al., 2023). Accordingly, the subsequent sections organize neuroimaging biomarkers across hierarchical levels of neural organization, spanning local activity, circuit-level interactions, large-scale network reconfiguration, and multimodal predictive modeling.

## 4 EEG biomarkers of NIBS effects in post-stroke recovery

**Fig. 2:**
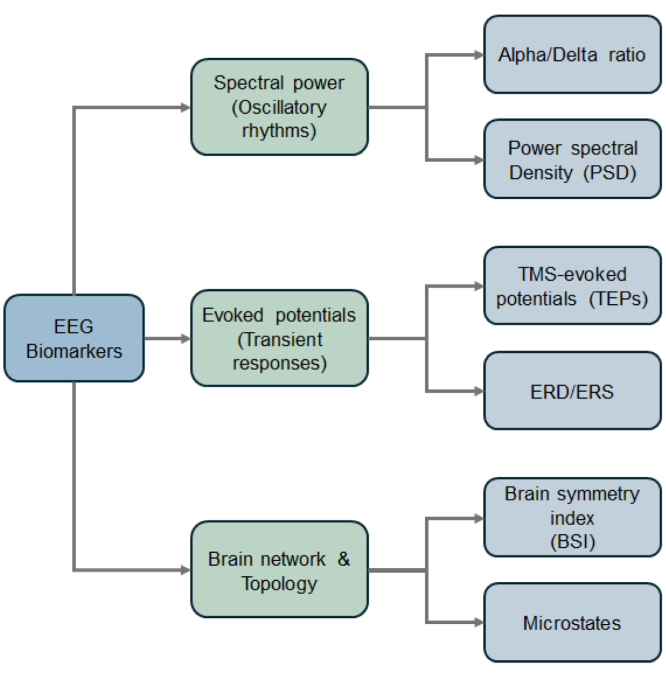
Hierarchical taxonomy of EEG biomarkers in post-stroke neurorehabilitation. EEG-derived markers are organized into three analytical domains: (1) Spectral power (oscillatory rhythms), including frequency-specific indices such as the alpha/delta ratio and power spectral density (PSD); (2) Evoked potentials (transient responses), encompassing TMS-evoked potentials (TEPs) and event-related desynchronization/synchronization (ERD/ERS); and (3) Brain Network & Topology, which evaluates systems-level reorganization using connectivity metrics (e.g., global efficiency, PDC), hemispheric asymmetry indices (e.g., BSI), and transient microstate dynamics. This taxonomy reflects a progression from local oscillatory activity to large-scale network reorganization.

EEG provides temporally precise markers of stimulation-induced neuroplasticity and enables multi-scale characterization of cortical dynamics in post-stroke recovery. As illustrated in Fig. 2, EEG biomarkers can be organized into three hierarchical domains reflecting distinct levels of neurophysiological organization: (1) local oscillatory activity, (2) circuit-level reactivity, and (3) large-scale network reorganization. This hierarchical framework captures the progression from baseline cortical state to perturbation responses and systems-level reconfiguration. Representative studies across these domains are summarized in Table 1.

**Table 1:** Summary of EEG, fMRI, and multimodal neuroimaging studies investigating NIBS effects in post-stroke recovery.

Domain	Study (Year)	Sample size	Task	NIBS intervention details			Outcomes & biomarker type		
		& design		Target (Anode/Cathode)	Protocol & parameters	Control condition	Neuroimaging biomarker	Clinical findings	Interactions
EEG	Chen et al. (2022)	n = 19, Crossover	Rest	Anode: Ipsilesional M1 Cathode: Contra. Supraorbital	tDCS (1 mA, 20 min)	Sham tDCS	α power modulation (Monitoring)	NR	Protocol-dep.
	Tscherpel et al. (2020)	n = 28, Parallel	Rest	Ipsilesional M1	TMS-EEG (110% RMT, 150 pulses)	Active (Vertex)	TEP responsivity (Predictive)	Improved (FMA-UE, r = 0.48)	Recovery predictor
	Kasashima-Shindo et al. (2015)	n = 18, Parallel	BCI	Anode: Ipsilesional M1 Cathode: Contra. Supraorbital	Anodal tDCS (1 mA, 10 min)	No-stim Control	mu-rhythm ERD (Monitoring)	Improved (FMA-UE, d = 0.64)	BCI efficacy
	Zhang et al. (2024)	n = 24, Parallel	Rest	Cerebellum	iTBS (80% RMT, 600 pulses)	Sham iTBS	Microstate Class C/D (Monitoring)	NR	Temporal stability
	Z. Wang et al. (2022)	n = 24, Parallel	Cog.	Anode: Left DLPFC Cathode: Right Supraorbital	Anodal tDCS (2 mA, 20 min)	Sham tDCS	θ power modulation (Monitoring)	Improved (MMSE, d = 0.52)	Executive link
	Liu et al. (2023)	n = 15, Parallel	Rest	Anode: Ipsilesional M1 Cathode: Contra. Supraorbital	Anodal tDCS (1.8 mA, 20 min)	Sham tDCS	δ↓,α↑, BFN (Monitoring)	Improved (FMA-UE, d = 0.58)	Network balance
	Yu et al. (2021)	n = 22, Parallel	Rest	Anode: Ipsilesional M1 Cathode: Contra. Supraorbital	Anodal tDCS (1 mA, 20 min)	Sham tDCS	β-band network (Monitoring)	NR	PDC analysis
	Bai et al. (2023a)	n = 20, Crossover	Rest	Ipsilesional M1	iTBS (80% AMT, 600 pulses)	Sham iTBS	P30/N45 TEPs (Monitoring)	NR	Local excitability
	Ding et al. (2022)	n = 22, Parallel	Rest	Ipsilesional M1	iTBS (70% RMT, 1200 pulses)	Sham iTBS	Nat. Freq. norm. (Monitoring)	NR	Ipsilesional M1
	Bai et al. (2023b)	n = 23, Parallel	Rest	Ipsilesional M1	TMS-EEG (110% RMT, 100 pulses)	Sham TMS	Intracortical net. (Predictive)	NR	Perturbation spread
	Brancaccio et al. (2025)	n = 19, Crossover	Rest	Bilateral M1	EEG-TMS (110% RMT, 120 pulses)	Sham TMS	Phase-dep. states (Predictive)	NR	State-dependent
	Mane et al. (2019)	n = 19, Parallel	BCI	Anode: Ipsilesional M1 Cathode: Contra. Supraorbital	Anodal tDCS (1 mA, 20 min)	Active tDCS	BSI, PRI indices (Predictive)	Improved (FMA-UE, r = 0.55)	Prognostic value
	Xu et al. (2021)	n = 24, Parallel	Visual	Occipital Lobe	tACS + tDCS (1.5 mA, 20 min)	Sham tACS	FCN reorganization (Monitoring)	Improved (Visual Field, d = 0.61)	Vision restoration
	Ding et al. (2021)	n = 30, Parallel	Rest	Ipsilesional M1	iTBS (70% RMT, 1200 pulses)	Sham iTBS	Global efficiency (Monitoring)	NR	Network topology
	Liu et al. (2024)	n = 36, Parallel	Mirror	Anode: Ipsilesional M1 Cathode: Contra. Supraorbital	tDCS (1 mA, 20 min)	Sham tDCS	Gating-by-inhib. (Monitoring)	Improved (FMA-UE, d = 0.70)	Synergistic effect
	C. Wang et al. (2022)	n = 32, Crossover	Rest	Anode: Ipsilesional M1 Cathode: Contra. Supraorbital	tDCS (1 mA, 20 min)	Sham tDCS	ERD/ERS patterns (Monitoring)	NR	Lesion dependency

	Xie et al. (2022)	n = 18, Parallel	Speech	Left IFG/Right Supraorbital	Theta-tACS (6 Hz, 1 mA, 20 min)	Sham tACS	Theta oscillation entrainment (Predictive)	Improved (WAB, d = 0.55)	Speech recovery
	Schuhmann et al. (2022)	n = 16, Crossover	Atten.	Contralesional PPC (HD)	Alpha-tACS (10 Hz, 1.5 mA, 20 min)	Sham tACS	Alpha power restoration (Monitoring)	Improved (Visual Bias, $\eta_{p}^{2}$= 0.34)	Neglect reduction
	Wu et al. (2016)	n = 40, Parallel	Rest	Bilateral Mastoids	tACS (20 Hz, 0.4 mA, 30 min)	Conventional Therapy	Intracranial hemodynamics (Monitoring)	Improved (NIHSS, d = 0.72)	Hemodynamic link
Naros and Gharabaghi (2017)	n = 14, Parallel	BMI	Ipsilesional M1/Supraorbital	Beta-tACS (20 Hz, 1 mA, 20 min)	Sham tACS	Corticomotor excitability (Predictive)	Improved (BMI Accuracy, d = 0.62)	Self-regulation

fMRI	Li et al. (2016)	n = 12, Parallel	Rest	Ipsilesional M1	5 Hz rTMS (120% RMT, 1000 pulses/day, 10 days)	Sham rTMS	Ipsilesional M1 FC (Monitoring)	Improved motor recovery	Functional reorg.
	Li et al. (2020)	n = 30, Parallel	Rest	Left DLPFC	5 Hz rTMS (20 min/day, 3 weeks)	Sham/control	fALFF + FC (Monitoring)	Improved MMSE/MoCA	PSCI modulation
	Yin et al. (2020)	n = 34, Parallel	Rest	Left DLPFC	10 Hz rTMS (80% RMT, 2000 pulses)	No-stim control	ALFF + FC (Monitoring)	Improved cognition/ADL	Frontal activity
	Zou et al. (2025)	n = 170, Parallel	Rest	Left DPC/DLPFC	5 Hz rTMS (90% RMT, 30 min, 30 days)	Sham rTMS	Motor-region FC/FCA (Monitoring)	Improved WMFT/FMA	DLPFC–motor FC
	Guo et al. (2021)	n = 33, Parallel	Rest	Ipsilesional/Contralesional M1	10 Hz vs. 1 Hz rTMS (90% RMT, 1500 vs. 900 pulses)	Sham rTMS	Motor network FC (Monitoring)	Improved FMA/BI/NIHSS	Frequency effect
	Hsu et al. (2023)	n = 27, Parallel	Rest	Bilateral M1	Bihemispheric tDCS (2 mA, 20 min)	Sham tDCS	Sensorimotor ROI-FC (Predictive/Monitoring)	Improved FMA-UE	FC-response assoc.
	Cherney et al. (2021)	n = 12, Parallel	Language	Perilesional cortex	tDCS (1 mA, 13 min)	Sham tDCS	Task-related BOLD activation (Monitoring)	Improved language function	Aphasia recovery
	Qin et al. (2021)	n = 41, Parallel	Rest	Contralesional M1	1 Hz rTMS (90% MT, 1200 pulses)	Sham rTMS	ICA + dFC (Monitoring)	FMA-related network change	Temporal dynamics
	Lee et al. (2018)	n = 24, Parallel	Rest	Bilateral M1	Dual-mode stimulation (10 Hz rTMS + 2 mA tDCS)	rTMS-only control	Interhemispheric FC + GE (Monitoring)	Improved motor recovery	Interhemispheric balance
	Chiu et al. (2020)	n = 30, Parallel	Motor	Multifocal cortical sites	TRPMS (40 min, 20 sessions)	Sham TRPMS	Task-fMRI BOLD voxels (Safety/Monitoring)	Safe; motor improvement trend	Cortical reorg.
	Olgiati et al. (2024)	n = 22, Crossover	Atten.	Right DLPFC	HD-tDCS (1 mA, 10 min)	Sham tDCS	Whole-brain FC (Monitoring)	Improved target detection	Contralesional networks
	Zhong et al. (2025)	n = 65 randomized; n = 53 analyzed	Rest	DLPFC	tDCS (active vs. sham)	Sham tDCS	FC + graph topology (Monitoring)	Improved MMSE/MoCA	Network integration
	Liu et al. (2025)	n = 64, Parallel	Rest	Non-lesioned M1	1 Hz rTMS (90% rMT, 1200 pulses, 8 weeks)	No-rTMS control	Switching rate (Predictive/Monitoring)	Improved FMA-UE/MBI	Dynamic flexibility
	Chen et al. (2021)	n = 13, Crossover	Rest	Ipsilesional M1 Return: Contra. supraorbital ridge	tACS (10/20 Hz, 1 mA, 20 min)	Sham tACS	Graph topology (Monitoring)	NR	Frequency-specific modulation

Multi	Jia et al. (2025)	n = 26, Parallel	MI	Mixed M1 sites	10 Hz/1 Hz rTMS (100% RMT, 600 pulses/session, 10 sessions)	Sham rTMS	EEG cortical activation patterns (Predictive)	Improved (MI decoding/FMA subgroup)	MI decoding
	Salazar et al. (2024)	n = 20 stroke, Parallel	Motor	Motor cortex	Concurrent tDCS-fMRI (2 mA, 10 min)	Sham tDCS	FC + E-field + DAN topology (Monitoring/Predictive)	Improved (FMA-UE)	Attention-network link
	Yuan et al. (2023)	n = 25, Parallel	Rest	Anode: Ipsilesional M1 Cathode: Contra. supraorbital	Anodal tDCS (1 mA, 20 min)	Sham tDCS	E-field prediction of FC change (Predictive)	NR	Comp. modeling
	(2017) Darkow et al. (2017)	n = 16, Crossover	Name	Left M1	Anodal tDCS (1 mA, 20 min)	Sham tDCS	Task BOLD + ICA language network (Monitoring)	Acute neural modulation	Task-NIBS interact.
	Yuan et al. (2022)	n = 13, Crossover	Rest/Task	Ipsilesional M1 Return: Contra. supraorbital ridge	tACS (10/20 Hz, 1 mA, 20 min)	Sham tACS	Task activation + seed-based FC + graph theory (Monitoring)	NR	Frequency tuning

**Table note.** The table reports study design, experimental state or task, stimulation target, stimulation protocol, control condition, primary neuroimaging biomarker, clinical or behavioral finding, and inferred mechanistic interpretation. Task/state labels indicate the experimental context in which neuroimaging biomarkers were acquired. *Rest* refers to resting-state recordings acquired without an explicit behavioral task; *Motor* refers to overt upper-limb or hand movement paradigms; *MI* refers to motor imagery; *Name* refers to picture naming or language production tasks; *Atten.* refers to attention or neglect-related behavioral paradigms; *Cog.* refers to cognitive assessment or cognitive training paradigms; and *Rest/Task* indicates studies combining resting-state and task-based neuroimaging.

**Abbreviations:** ADL, activities of daily living; ALFF, amplitude of low-frequency fluctuations; ARAT, Action Research Arm Test; BOLD, blood oxygen-level dependent; BI, Barthel Index; CST, corticospinal tract; DAN, dorsal attention network; dFC, dynamic functional connectivity; DLPFC, dorsolateral prefrontal cortex; DPC, dorsolateral prefrontal cortex; EEG, electroencephalography; EF, electric field; FC, functional connectivity; FCA, functional connectivity analysis; fALFF, fractional amplitude of low-frequency fluctuations; FMA, Fugl-Meyer Assessment; FMA-UE, Fugl-Meyer Assessment of Upper Extremity; GE, global efficiency; HD-tDCS, high-definition transcranial direct current stimulation; ICA, independent component analysis; MBI, Modified Barthel Index; MEP, motor-evoked potential; MI, motor imagery; MMSE, Mini-Mental State Examination; MoCA, Montreal Cognitive Assessment; M1, primary motor cortex; NIHSS, National Institutes of Health Stroke Scale; NIBS, non-invasive brain stimulation; NR, not reported; PLV, phase-locking value; PMC, premotor cortex; PSCI, post-stroke cognitive impairment; ReHo, regional homogeneity; ROI, region of interest; rMT/RMT, resting motor threshold; rs-fMRI, resting-state functional magnetic resonance imaging; rTMS, repetitive transcranial magnetic stimulation; SMN, sensorimotor network; tACS, transcranial alternating current stimulation; tDCS, transcranial direct current stimulation; TRPMS, transcranial rotating permanent magnet stimulation; WAB-AQ, Western Aphasia Battery Aphasia Quotient; WMFT, Wolf Motor Function Test.

### 4.1 Local oscillatory activity

TMS–EEG provides a direct, muscle-independent measure of cortical excitability in the post-stroke brain. TMS-evoked potentials (TEPs) capture immediate cortical responses to perturbation, enabling assessment of both local reactivity and effective signal propagation (Tscherpel et al., 2020). Reduced ipsilesional TEP responsivity is associated with impaired recovery, whereas restoration of early components reflects rebalancing of excitatory–inhibitory dynamics (Tscherpel et al., 2020). Intermittent theta burst stimulation (iTBS) rapidly modulates intracortical circuits, particularly early components such as P30 and N45, which are linked to glutamatergic and GABAergic mechanisms (Bai et al., 2023a).

Cortical reactivity is strongly state-dependent. The phase of ongoing sensorimotor oscillations influences TEP amplitude and propagation, indicating that stimulation efficacy depends on baseline neural state (Brancaccio et al., 2025). In addition, NIBS can normalize intrinsic oscillatory frequency in the ipsilesional motor cortex, counteracting pathological slowing after stroke (Ding et al., 2022). These perturbation-based metrics therefore provide mechanistically grounded biomarkers of circuit-level plasticity beyond peripheral motor output.

### 4.2 Circuit-level reactivity

Frequency-specific oscillatory activity represents a sensitive monitoring biomarker of cortical state and stimulation-induced plasticity. Post-stroke EEG commonly exhibits pathological slowing, characterized by increased delta activity and reduced alpha power in the ipsilesional hemisphere. NIBS modulates these rhythms in a polarity- and protocol-dependent manner; for example, anodal tDCS has been associated with enhancement of ipsilesional alpha power, suggesting partial normalization of cortical excitability (Chen et al., 2022). Task-related oscillatory markers further capture functional engagement of motor circuits. Enhancement of mu-rhythm event-related desynchronization (ERD) following anodal stimulation has been linked to improved performance in BCI-assisted rehabilitation (Kasashima-Shindo et al., 2015), although ERD/ERS modulation is influenced by lesion topology and stimulation parameters (C. Wang et al., 2022). Beyond static direct current modulations, tACS offers a frequency-specific mechanism to directly entrain target cortical rhythms and probe circuit responsivity. For instance, theta-frequency tACS (6 Hz) applied over the left inferior frontal gyrus has been demonstrated to enhance speech comprehension in chronic post-stroke aphasia by directly modulating language-related oscillatory networks (Xie et al., 2022). Similarly, high-definition alpha-tACS (10 Hz) targeted at the contralesional posterior parietal cortex significantly mitigates hemispatial neglect symptoms, highlighting the utility of frequency-tuned oscillatory markers as indicators of interhemispheric balance restoration (Schuhmann et al., 2022).

Oscillatory biomarkers also capture neurophysiological processes associated with cognitive reorganization. Modulation of frontal theta power following dorsolateral prefrontal cortex stimulation correlates with improvements in executive function (Z. Wang et al., 2022). Quantitative EEG-derived indices such as the Brain Symmetry Index (BSI) and Power Ratio Index

(PRI) demonstrate utility as predictive biomarkers for responsiveness to tDCS-based interventions (Mane et al., 2019). Moreover, theory-driven metrics grounded in the gating-by-inhibition framework provide mechanistic insight into how inhibitory oscillations regulate information flow during combined neuromodulation and behavioral therapy (Liu et al., 2024). These oscillatory signatures therefore constitute monitoring biomarkers that link local cortical state to behavioral recovery.

### 4.3 Network reorganization and topology

At the systems level, EEG-derived connectivity and graph-theoretical metrics quantify large-scale reorganization of post-stroke brain networks. Measures such as global efficiency and small-worldness capture integration–segregation balance within functional networks and have been shown to improve following excitatory stimulation protocols (Liu et al., 2023, Ding et al., 2021). Directional connectivity analyses further delineate information flow within reorganizing motor circuits; enhancement of beta-band connectivity assessed via Partial Directed Coherence (PDC) indicates strengthened inter-regional communication after tDCS (Yu et al., 2021). Concurrent TMS–EEG studies extend this framework by demonstrating how local perturbations propagate through distributed cortical networks, providing causal evidence of circuit-level reconfiguration (Bai et al., 2023b).

Network-level modulation is not restricted to motor systems. In visual domains, combined tACS–tDCS protocols facilitate connectivity reorganization associated with functional restoration following occipital stroke (Xu et al., 2021). Beyond static connectivity metrics, microstate analysis captures rapid transitions between quasi-stable global brain states. Modulation of specific microstate classes following NIBS reflects dynamic reconfiguration of large-scale cortical networks and highlights the importance of temporal stability in recovery processes (Zhang et al., 2024). These network-level biomarkers extend the characterization of post-stroke neuroplasticity from local cortical excitability to distributed circuit integration and temporal network organization. Furthermore, systems-level network configurations can be modulated by rhythmic entrainment protocols across diverse electrode configurations. The application of 20 Hz tACS over bilateral mastoids has been shown to optimize intracranial hemodynamics and facilitate subacute clinical recovery, outlining a functional bridge between alternating electric fields and macro-scale vascular plastic changes (Wu et al., 2016). Within motor neurorehabilitation frameworks, beta-frequency tACS (20 Hz) integrated with brain-machine interface (BMI) training environments serves as an effective interventional tool to bolster corticomotor excitability and promote autonomous brain self-regulation kinetics (Naros and Gharabaghi, 2017).

## 5 fMRI biomarkers of NIBS effects in post-stroke recovery

**Fig. 3:**
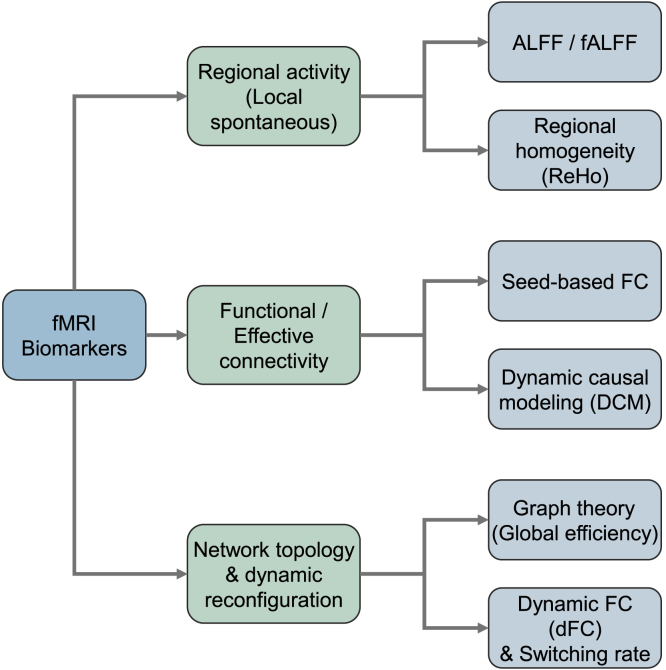
Hierarchical taxonomy of fMRI biomarkers in post-stroke neurorehabilitation. Representative examples of commonly used fMRI biomarkers are organized into three analytical levels based on spatial and network complexity: (1) Regional BOLD activity, reflecting local spontaneous and task-related activity patterns, including amplitude of low-frequency fluctuations (ALFF/fALFF) and regional homogeneity (ReHo); (2) Functional and effective connectivity, characterizing inter-regional interactions using approaches such as seed-based functional connectivity and dynamic causal modeling (DCM); (3) Network topology and dynamic reconfiguration, encompassing graph-theoretical metrics (e.g., global efficiency) and time-varying connectivity measures such as dynamic functional connectivity (dFC) and network switching rates. Additional analytical frameworks discussed in the text include task-based activation mapping, voxel-wise and ROI-to-ROI connectivity analyses, Independent Component Analysis (ICA), atlas-based network modeling, and precision functional mapping. This hierarchical framework maps fMRI biomarkers from local activity to systems-level connectomic reorganization.

fMRI provides spatially resolved biomarkers that capture multiple levels of post-stroke neuroplasticity, ranging from regional activity and inter-regional connectivity to large-scale network organization following NIBS. Whereas EEG primarily characterizes rapid electrophysiological processes with high temporal resolution, fMRI provides spatially resolved measures of regional activity and large-scale network organization. These complementary modalities provide a comprehensive framework for assessing NIBS-induced neuroplasticity across multiple spatial and temporal scales. As illustrated in Fig. 3, fMRI measures can be hierarchically organized into three analytical domains: (1) regional BOLD activity, (2) functional and effective connectivity, and (3) advanced network dynamics. This multi-scale framework parallels the electrophysiological taxonomy presented for EEG, spanning multiple levels of neural organization from local BOLD fluctuations to distributed connectomic reorganization.

The studies summarized in Table 1 demonstrate that NIBS-induced recovery is consistently associated with measurable changes in these fMRI-derived indices across motor, cognitive, and language domains. The following sections synthesize current evidence according to this hierarchical structure, emphasizing how regional activation patterns, connectivity remodeling, and dynamic network transitions contribute to biomarker-driven precision neuromodulation.

### 5.1 Regional BOLD activity

Regional fMRI biomarkers can be broadly categorized into resting-state and task-evoked activity measures. Resting-state fMRI captures spontaneous neural activity in the absence of explicit task demands, whereas task-based paradigms quantify regional activation during motor, language, or cognitive processes.

Within resting-state analyses, amplitude-based and local synchrony-based metrics are commonly employed. Amplitude of low-frequency fluctuations (ALFF), fractional ALFF (fALFF), and regional homogeneity (ReHo) quantify spontaneous BOLD fluctuations and local synchronization, providing sensitive markers of regional neuroplasticity after stroke. Excitatory stimulation protocols have been associated with increased ALFF, fALFF, and ReHo within ipsilesional motor and cognitive control regions, with these changes frequently paralleling behavioral recovery (Li et al., 2016, Li et al., 2020, Yin et al., 2020, Zou et al., 2025).

Complementing resting-state measures, task-based fMRI directly characterizes regional BOLD activation during behavioral execution. Neuromodulation-induced changes in activation volume, active voxel recruitment, and task-evoked cortical responses have been observed during motor execution, language production, and attentional paradigms (Cherney et al., 2021, Chiu et al., 2020, Olgiati et al., 2024, Darkow et al., 2017, Yuan et al., 2022). These task-related activation measures provide direct insight into how stimulation modifies the functional recruitment of surviving cortical systems and contributes to behavioral improvement after stroke (Chiu et al., 2020, Darkow et al., 2017).

### 5.2 Functional and effective connectivity

Functional connectivity analyses assess statistical dependencies between spatially distributed brain regions and provide important markers of post-stroke network reorganization. Many stroke neuromodulation studies have employed seed-based connectivity analyses to quantify stimulation-induced changes between predefined cortical targets and distributed brain regions, particularly within motor, language, and cognitive control networks (Hsu et al., 2023, Yuan et al., 2023, Guo et al., 2021, Lee et al., 2018).

However, connectivity investigations extend beyond conventional seed-based frameworks. Whole-brain voxel-wise connectivity analyses, ROI-to-ROI network models, and large-scale interhemispheric connectivity approaches have increasingly been used to characterize distributed reorganization patterns following stimulation (Hsu et al., 2023, Lee et al., 2018, Zhong et al., 2025, Salazar et al., 2024). These methods capture distributed interaction patterns and network-level reorganization that cannot be fully represented by a limited set of predefined seeds.

Connectivity may also be examined in a task-dependent manner, providing complementary information to conventional resting-state analyses. Approaches such as psychophysiological interaction (PPI) analysis can evaluate how stimulation influences context-dependent communication among distributed brain regions during behavioral performance. Although such methods remain relatively uncommon in the current stroke neuromodulation literature, they represent a promising direction for understanding stimulation-induced network reorganization under active task demands. Together, resting-state and task-based connectivity frameworks provide a more comprehensive characterization of how NIBS reshapes large-scale brain communication after stroke.

### 5.3 Network topology and dynamic reconfiguration

Network-level analyses extend beyond pairwise connectivity and aim to characterize the large-scale organization of functional brain systems. Data-driven Independent Component Analysis (ICA) has been used to identify distributed functional networks without requiring predefined seed regions, enabling characterization of stimulation-related changes in motor, language, attentional, and cognitive control systems (Qin et al., 2021, Darkow et al., 2017). Complementarily, atlas-based network modeling uses anatomical or functional parcellation schemes, such as the Automated Anatomical Labeling (AAL) atlas, to construct whole-brain connectivity matrices and quantify large-scale network architecture and inter-regional interactions (Lee et al., 2018, Zhong et al., 2025). These approaches provide complementary frameworks for characterizing distributed network organization after stroke.

Building upon these network representations, graph-theoretical analyses quantify topological properties of large-scale brain systems. Metrics such as global efficiency, clustering coefficient, modularity, and small-world organization provide measures of information integration and segregation across distributed networks. Recent studies have demonstrated that neuromodulation can enhance network efficiency and partially restore disrupted topological organization following stroke (Zhong et al., 2025, Chen et al., 2021).

While graph-theoretical analyses characterize the overall topology of brain networks, dynamic functional connectivity (dFC) captures temporal fluctuations in network interactions and reveals stimulation-induced normalization of abnormal brain-state transitions after stroke (Qin et al., 2021, Liu et al., 2025). ICA, atlas-based modeling, graph-theoretical approaches, and dFC provide complementary perspectives on large-scale functional reorganization. Emerging individualized approaches, including precision functional mapping, may further refine network-targeted neuromodulation by identifying subject-specific functional architectures and represent promising directions for future biomarker-guided stimulation strategies.

## 6 Multimodal integration and computational modeling of NIBS effects

### 6.1 Concurrent neurostimulation and real-time network modulation

EEG and fMRI provide distinct but complementary perspectives on NIBS-induced neuroplasticity. Integrating these modalities offers opportunities to link fast electrophysiological dynamics with systems-level network reorganization. Simultaneous integration of NIBS with fMRI enables direct observation of stimulation-induced modulation of distributed brain networks in vivo. Concurrent tDCS-fMRI studies indicate that behavioral improvement after stroke is not solely explained by local motor cortical activation, but may depend on large-scale network organization. For example, concurrent tDCS-fMRI demonstrated that stimulation-related motor improvement was most strongly associated with dorsal attention network topology and broader connectivity reorganization involving supplementary motor, inferior frontal, and temporo-occipital regions (Salazar et al., 2024).

Task-based concurrent stimulation further demonstrates that NIBS can acutely modulate residual task-relevant networks. In post-stroke aphasia, anodal tDCS delivered during picture-naming fMRI reduced activity in domain-general cognitive control regions while selectively enhancing language-network activity identified through ICA-based analyses, suggesting more efficient recruitment of residual language circuits during stimulation (Darkow et al., 2017). Frequency-specific stimulation effects are also observable during concurrent tACS-fMRI acquisition. In chronic stroke, 10 Hz and 20 Hz tACS applied over ipsilesional M1 induced distinct patterns of task-evoked activation, seed-based functional connectivity, and graph-theoretical network reorganization, supporting the importance of frequency-specific stimulation for modulating large-scale network organization after stroke (Yuan et al., 2022).

### 6.2 Computational modeling and predictive neuromodulation

Addressing inter-individual variability requires individualized computational modeling and network-informed characterization of stimulation susceptibility. Stroke lesions and anatomical heterogeneity can alter current flow and functional response profiles, emphasizing the importance of lesion-informed head models for stimulation optimization. Individualized electric-field modeling has shown that anodal tDCS over ipsilesional M1 facilitates functional connectivity within the ipsilesional sensorimotor network, and that the electric-field component normal to the ipsilesional M1 surface predicts individual functional connectivity changes following stimulation (Yuan et al., 2023).

Beyond electric-field modeling, variability in stimulation response is also influenced by pre-existing functional architecture and network organization. Baseline cortical activation patterns measured during motor imagery can influence the effectiveness of rTMS-induced motor imagery decoding enhancement, suggesting that individualized neural reorganization patterns should guide neuromodulation protocols (Jia et al., 2025). These findings highlight the importance of incorporating both structural and functional biomarkers when designing personalized stimulation strategies.

Integrative frameworks that combine regional activation patterns, functional connectivity, electrophysiological activity, and computational modeling provide a foundation for biomarker-driven, individualized, and eventually closed-loop neuromodulation strategies (Ovadia-Caro et al., 2019). Such approaches may improve patient stratification, optimize stimulation targeting, and facilitate adaptive interventions that dynamically respond to evolving patterns of post-stroke recovery.

**Table 2:** Neuroimaging biomarkers of NIBS and their translational implications for personalized neuromodulation.

Modality	Biomarker scale	Neurobiological interpretation	Clinical implication
EEG	Local activity (PSD, α/δ, natural frequency)	Cortical excitability and oscillatory balance	Selection of excitatory vs. inhibitory stimulation and frequency tuning
	Circuit reactivity (TEPs, ERD/ERS)	Intracortical excitation–inhibition balance and task engagement	Prediction of stimulation responsiveness and timing optimization
	Network topology (BSI, microstates)	Interhemispheric imbalance and temporal network flexibility	Bihemispheric targeting and recovery monitoring

fMRI	Regional activity (ALFF, ReHo)	Local metabolic demand and spontaneous synchronization	Identification of perilesional re-engagement and target localization
	Connectivity (FC, DCM)	Inter-regional coupling and directional information flow	Network-informed stimulation targeting and patient stratification
	Network dynamics (dFC, global efficiency, switching rate)	Time-varying network stability and integration capacity	Adaptive stimulation design and outcome prediction

Multimodal	Electric field modeling	Individualized current distribution and dose estimation	Dose optimization based on lesion anatomy
	EEG–fMRI integration	Multi-scale circuit profiling and predictive modeling	Development of closed-loop precision neuromodulation

## 7 Clinical translation toward personalized neuromodulation

The shift from standardized stimulation protocols to individualized neuromodulation is central to advancing stroke rehabilitation. Substantial inter-individual variability in treatment response necessitates biomarker-guided stratification based on functional network state and structural integrity. The complementary strengths of EEG and fMRI enable comprehensive characterization of rapid electrophysiological dynamics and large-scale network organization, thereby supporting biomarker-guided patient stratification and individualized stimulation planning. Integration of EEG and fMRI metrics enables identification of optimal stimulation targets, frequencies, and intensities tailored to each patient’s neurobiological profile. Precise targeting is particularly critical in tES, as stimulation effects strongly depend on electrode placement and the resulting modulation of corticomotor excitability (Lee et al., 2015). Table 2 organizes neuroimaging biomarkers according to modality, network scale, and mechanistic significance, linking each metric to its translational implication. This structured mapping illustrates how biomarker-driven stratification can guide target selection, stimulation parameters, and adaptive neuromodulation strategies in clinical practice.

Functional navigation and structural constraints jointly refine therapeutic precision. fMRI-guided targeting of perilesional language cortex improves naming performance in post-stroke aphasia, demonstrating the benefit of task-specific network localization (Cherney et al., 2021). Structural biomarkers such as corticospinal tract lesion load further predict motor recovery potential and inform candidate selection for bihemispheric stimulation (Hsu et al., 2023). BOLD-monitored multifocal stimulation confirms that distributed cortical engagement can be achieved safely, supporting circuit-level modulation strategies (Chiu et al., 2020). Complementary electric field modeling provides individualized dose estimation based on lesion anatomy and cortical morphology.

The long-term objective is adaptive closed-loop neuromodulation in which real-time EEG or fMRI biomarkers dynamically guide stimulation parameters. Achieving this goal requires harmonized biomarker pipelines, validation of predictive models in large cohorts, and integration of AI-driven decision frameworks into clinical workflows. Bridging mechanistic biomarker discovery with scalable therapeutic deployment will define the next stage of precision neuromodulation.

## 8 Challenges and future directions

Despite the therapeutic promise of NIBS, several critical challenges must be resolved before it can be established as a standardized clinical intervention in stroke rehabilitation. A major obstacle remains the pronounced inter-individual variability in treatment response, influenced by lesion topography, chronicity, baseline functional state, and anatomical determinants of electric field distribution. As a result, uniform stimulation protocols frequently yield inconsistent outcomes. The development and large-scale validation of robust neuroimaging biomarkers capable of stratifying patients and predicting stimulation responsiveness therefore remains an essential priority.

A second challenge concerns the technical and logistical complexity of integrating multimodal biomarkers into routine practice. Although the combination of EEG, fMRI, and computational modeling provides detailed insight into circuit-level plasticity, the financial cost, computational burden, and requirement for specialized expertise limit widespread clinical implementation. Future progress will depend on scalable solutions, including streamlined acquisition pipelines, wearable EEG-integrated stimulation systems, and practical electric field modeling frameworks. In parallel, the field must advance from focal region-based targeting toward circuit-level and network-based neuromodulation strategies that account for distributed connectivity patterns.

Advancing toward adaptive closed-loop neuromodulation will require robust real-time electrophysiological and hemodynamic biomarkers capable of guiding stimulation parameters according to evolving neural states. Integration of AI-driven predictive models trained on large, multicenter datasets may further enable longitudinal personalization across different stages of stroke recovery. At the same time, sustained clinical translation demands careful consideration of data governance, long-term effects of chronic brain modulation, and equitable access to emerging technologies. Addressing these scientific, technical, and societal challenges will be essential to transform precision neuromodulation from an experimental framework into a scalable and sustainable component of stroke rehabilitation. Although scalp EEG remains the dominant modality in post-stroke NIBS research, invasive electrophysiological recordings may provide future opportunities for higher-resolution characterization of stimulation-induced mechanisms. The present synthesis primarily focused on rTMS, tDCS, and emerging tACS paradigms; however, additional stimulation approaches such as tRNS warrant further investigation because of their potential relevance to oscillatory modulation and biomarker-guided rehabilitation.

## 9 Conclusion

Neuroimaging biomarkers provide critical mechanistic and translational insights into NIBS-induced neuroplasticity following stroke. EEG and fMRI capture complementary dimensions of cortical reorganization spanning local excitability, circuit-level dynamics, and large-scale network remodeling. Rather than serving as interchangeable biomarkers, EEG and fMRI provide complementary information across temporal and spatial scales. The conceptual framework proposed in this review provides a structured perspective on how modality-specific biomarkers can be integrated to characterize neuroplasticity, explain inter-individual variability in treatment response, and guide biomarker-driven personalized neuromodulation. Integrating multimodal biomarkers with computational modeling and individualized stimulation strategies may enable biomarker-driven precision neuromodulation frameworks for post-stroke rehabilitation. Continued validation across larger and more diverse populations will be essential for translating these approaches into scalable clinical practice.

## CRediT authorship contribution statement

**Seoyeon Kim:** Writing – original draft, Methodology, Investigation, Formal analysis, Data curation. **Sang Wook Lee:** Writing – review & editing, Conceptualization. **Minji Lee:** Writing – review & editing, Supervision, Methodology, Conceptualization.

## Declaration of competing interest

The authors declare that they have no known competing financial interests or personal relationships that could have appeared to influence the work reported in this paper.

## Data Availability

No data was used for the research described in the article.

## References

[b1] Bai Z., Zhang J.J., Fong K.N.K. (2023). Immediate effects of intermittent theta burst stimulation on primary motor cortex in stroke patients: A concurrent TMS-EEG study. IEEE Trans. Neural Syst. Rehabil. Eng..

[b2] Bai Z., Zhang J.J., Fong K.N.K. (2023). Intracortical and intercortical networks in patients after stroke: A concurrent TMS-EEG study. J. Neuroeng. Rehabil..

[b3] Bajaj S., Butler A.J., Drake D., Dhamala M. (2015). Brain effective connectivity during motor-imagery and execution following stroke and rehabilitation. Neuroimage Clin..

[b4] Brancaccio A., Tabarelli D., Baur D., Roesch J., Mahmoud W., Ziemann U., Belardinelli P. (2025). Motor cortex excitability states in chronic stroke patients probed by EEG-TMS. Clin. Neurophysiol..

[b5] Chen Y., Wang C., Song P., Sun C., Zhang Y., Zhao X., Du J. (2022). Alpha rhythm of electroencephalography was modulated differently by three transcranial direct current stimulation protocols in patients with ischemic stroke. Front. Hum. Neurosci..

[b6] Chen C., Yuan K., Chu W.C.-w., Tong R.K.-y. (2021). The effects of 10 Hz and 20 Hz tACS in network integration and segregation in chronic stroke: A graph theoretical fMRI study. Brain Sci..

[b7] Cherney L.R., Babbitt E.M., Wang X., Pitts L.L. (2021). Extended fMRI-guided anodal and cathodal transcranial direct current stimulation targeting perilesional areas in post-stroke aphasia: A pilot randomized clinical trial. Brain Sci..

[b8] Chiu D., McCane C.D., Lee J., John B., Nguyen L., Butler K., Gadhia R., Misra V., Volpi J.J., Verma A., Helekar S.A. (2020). Multifocal transcranial stimulation in chronic ischemic stroke: A phase 1/2a randomized trial. Brain Stimul..

[b9] Darkow R., Martin A., W”urtz A., Fl”oel A., Meinzer M. (2017). Transcranial direct current stimulation effects on neural processing in post-stroke aphasia. Hum. Brain Mapp..

[b10] Dayan E., Censor N., Buch E.R., Sandrini M., Cohen L.G. (2013). Noninvasive brain stimulation: From physiology to network dynamics and rehabilitation. Nat. Neurosci..

[b11] Di Pino G., Pellegrino G., Assenza G., Capone F., Ferreri F., Formica D., Ranieri F., Tombini M., Ziemann U., Rothwell J.C. (2014). Modulation of brain plasticity in stroke: A novel model for neurorehabilitation. Nat. Rev. Neurol..

[b12] Ding Q., Chen S., Chen J., Zhang S., Peng Y., Chen Y., Chen J., Li X., Chen K., Cai G., Xu G., Lan Y. (2022). Intermittent theta burst stimulation increases natural oscillatory frequency in ipsilesional motor cortex post-stroke: A transcranial magnetic stimulation and electroencephalography study. Front. Aging Neurosci..

[b13] Ding Q., Zhang S., Chen S., Chen J., Li X., Chen J., Peng Y., Chen Y., Chen K., Cai G., Xu G., Lan Y. (2021). The effects of intermittent theta burst stimulation on functional brain network following stroke: An electroencephalography study. Front. Neurosci..

[b14] Guo Z., Jin Y., Bai X., Jiang B., He L., McClure M.A., Mu Q. (2021). Distinction of high- and low-frequency repetitive transcranial magnetic stimulation on the functional reorganization of the motor network in stroke patients. Neural Plast..

[b15] van den Heuvel M.P., Hulshoff Pol H.E. (2010). Exploring the brain network: A review on resting-state fMRI functional connectivity. Eur. Neuropsychopharmacol..

[b16] Hsu S.P., Lu C.F., Lin B.F., Tang C.W., Kuo I.J., Tsai Y.A., Guo C.Y., Lee P.L., Shyu K.K., Niddam D.M., Lee I.H. (2023). Effects of bihemispheric transcranial direct current stimulation on motor recovery in subacute stroke patients: A double-blind, randomized sham-controlled trial. J. Neuroeng. Rehabil..

[b17] Jia T., Mo L., McGeady C., Sun J., Liu A., Ji L., Xi J., Li C. (2025). Cortical activation patterns determine effectiveness of rTMS-induced motor imagery decoding enhancement in stroke patients. IEEE Trans. Biomed. Eng..

[b18] Kasashima-Shindo Y., Fujiwara T., Ushiba J., Matsushika Y., Kamatani D., Oto M., Ono T., Nishimoto A., Shindo K., Kawakami M., Tsuji T., Liu M. (2015). Brain-computer interface training combined with transcranial direct current stimulation in patients with chronic severe hemiparesis. J. Rehabil. Med..

[b19] Lee M., Kim Y.-H., Im C.-H., Kim J.-H., Park C.-h., Chang W.H., Lee A. (2015). What is the optimal anodal electrode position for inducing corticomotor excitability changes in transcranial direct current stimulation?. Neurosci. Lett..

[b20] Lee M., Kim Y.-H., Lee S.-W. (2022). Motor impairment in stroke patients is associated with network properties during consecutive motor imagery. IEEE Trans. Biomed. Eng..

[b21] Lee J., Park E., Lee A., Chang W.H., Kim D.S., Shin Y.I., Kim Y.H. (2018). Modulating brain connectivity by simultaneous dual-mode stimulation over bilateral primary motor cortices in subacute stroke patients. Neural Plast..

[b22] Li Y., Luo H., Yu Q., Yin L., Li K., Li Y., Fu J. (2020). Cerebral functional manipulation of repetitive transcranial magnetic stimulation in cognitive impairment patients after stroke: An fMRI study. Front. Neurol..

[b23] Li J., Zhang X.W., Zuo Z.T., Lu J., Meng C.L., Fang H.Y., Xue R., Fan Y., Guan Y.Z., Zhang W.H. (2016). Cerebral functional reorganization in ischemic stroke after repetitive transcranial magnetic stimulation: An fMRI study. CNS Neurosci. Ther..

[b24] Liu C.L., Su K.H., Horng Y.S., Chen C.L., Huang S.H., Wu C.Y. (2024). Theory-driven EEG indexes for tracking motor recovery and predicting the effects of hybridizing tDCS with mirror therapy in stroke patients. IEEE Trans. Neural Syst. Rehabil. Eng..

[b25] Liu X., Wang X., Zhuang X., Qiu S., Tang Y., Qin Y. (2025). Transcranial magnetic stimulation regulates brain functional network dynamics in stroke patients: A randomised controlled trial. J. Neuroeng. Rehabil..

[b26] Liu M., Xu G., Yu H., Wang C., Sun C., Guo L. (2023). Effects of transcranial direct current stimulation on EEG power and brain functional network in stroke patients. IEEE Trans. Neural Syst. Rehabil. Eng..

[b27] Mane R., Chew E., Phua K.S., Ang K.K., Robinson N., Vinod A.P., Guan C. (2019). Prognostic and monitory EEG-biomarkers for BCI upper-limb stroke rehabilitation. IEEE Trans. Neural Syst. Rehabil. Eng..

[b28] Naros G., Gharabaghi A. (2017). Physiological and behavioral effects of β-tACS on brain self-regulation in chronic stroke. Brain Stimul..

[b29] Olgiati E., Violante I.R., Xu S., Sinclair T.G., Li L.M., Crow J.N., Kapsetaki M.E., Calvo R., Li K., Nayar M., Grossman N., Patel M.C., Wise R.J.S., Malhotra P.A. (2024). Targeted non-invasive brain stimulation boosts attention and modulates contralesional brain networks following right hemisphere stroke. Neuroimage Clin..

[b30] Ovadia-Caro S., Khalil A.A., Sehm B., Villringer A., Nikulin V.V., Nazarova M. (2019). Predicting the response to non-invasive brain stimulation in stroke. Front. Neurol..

[b31] Palva S., Palva J.M. (2012). Discovering oscillatory interaction networks with M/EEG: Challenges and breakthroughs. Trends Cogn. Sci..

[b32] Qin Y., Liu X., Guo X., Liu M., Li H., Xu S. (2021). Low-frequency repetitive transcranial magnetic stimulation restores dynamic functional connectivity in subcortical stroke. Front. Neurol..

[b33] Salazar C.A., Welsh J.M., Lench D., Harmsen I.E., Jensen J.H., Grewal P., Yazdani M., Al Kasab S., Spiotta A., Bonilha L., George M.S., Kautz S.A., Rowland N.C. (2024). Concurrent tDCS-fMRI after stroke reveals link between attention network organization and motor improvement. Sci. Rep..

[b34] Schuhmann T., Duecker F., Middag-van Spanje M., Gallotto S., van Heugten C., Schrijnemaekers A.-C., van Oostenbrugge R., Sack A.T. (2022). Transcranial alternating brain stimulation at alpha frequency reduces hemispatial neglect symptoms in stroke patients. Int. J. Clin. Health Psychol..

[b35] Tscherpel C., Dern S., Hensel L., Ziemann U., Fink G.R., Grefkes C. (2020). Brain responsivity provides an individual readout for motor recovery after stroke. Brain.

[b36] Wang C., Chen Y., Song P., Yu H., Du J., Zhang Y., Sun C. (2022). Varied response of EEG rhythm to different tDCS protocols and lesion hemispheres in stroke subjects with upper limb dysfunction. Neural Plast..

[b37] Wang Z., Li J., Wang X., Liu S., Wu W. (2022). Effect of transcranial direct-current stimulation on executive function and resting EEG after stroke: A pilot randomized controlled study. J. Clin. Neurosci..

[b38] Wassermann E.M. (2002). Variation in the response to transcranial magnetic brain stimulation in the general population. Clin. Neurophysiol..

[b39] Wiethoff S., Hamada M., Rothwell J.C. (2014). Variability in response to transcranial direct current stimulation of the motor cortex. Brain Stimul..

[b40] Wu J.-f., Wang H.-j., Wu Y., Li F., Bai Y.-l., Zhang P.-y., Chan C.C. (2016). Efficacy of transcranial alternating current stimulation over bilateral mastoids (tACSbm) on enhancing recovery of subacute post-stroke patients. Top. Stroke Rehabil..

[b41] Xie X., Hu P., Tian Y., Wang K., Bai T. (2022). Transcranial alternating current stimulation enhances speech comprehension in chronic post-stroke aphasia patients: A single-blind sham-controlled study. Brain Stimul.: Basic, Transl. Clin. Res. Neuromodulation.

[b42] Xu J., Wu Z., Nürnberger A., Sabel B.A. (2021). Reorganization of brain functional connectivity network and vision restoration following combined tACS-tDCS treatment after occipital stroke. Front. Neurol..

[b43] Yin M., Liu Y., Zhang L., Zheng H., Peng L., Ai Y., Luo J., Hu X. (2020). Effects of rTMS treatment on cognitive impairment and resting-state brain activity in stroke patients: A randomized clinical trial. Front. Neural Circuits.

[b44] Yu H., Wang H., Liu M., Wang C., Sun C., Xu G., Guo L. (2021). Effects of transcranial direct current stimulation on stroke based on brain functional networks. IEEE Trans. Magn..

[b45] Yuan K., Chen C., Lou W.-T., Khan A., Ti E.C.-H., Lau C.C.-Y., Wang X., Chu W.C.-W., Tong R.K.-Y. (2022). Differential effects of 10 and 20 Hz brain stimulation in chronic stroke: A tACS-fMRI study. IEEE Trans. Neural Syst. Rehabil. Eng..

[b46] Yuan K., Ti C.E., Wang X., Chen C., Lau C.C.Y., Chu W.C.W., Tong R.K.Y. (2023). Individual electric field predicts functional connectivity changes after anodal transcranial direct-current stimulation in chronic stroke. Neurosci. Res..

[b47] Zhang H., Yang X., Yao L., Liu Q., Lu Y., Chen X., Wang T. (2024). EEG microstates analysis after TMS in patients with subacute stroke during the resting state. Cereb. Cortex.

[b48] Zhong J., Jing X., Liang Y., Hao P., Peng R., Xin R. (2025). The impact of transcranial direct current stimulation on brain network connectivity and topology in post-stroke cognitive impairment patients: A resting-state fMRI study. Neurol. Sci..

[b49] Zou X., Zhu J., Hu S., Hou Z., Ma G. (2025). Application of non-invasive brain stimulation combined with functional magnetic resonance imaging in post-stroke motor function rehabilitation. J. Neurosci. Methods.

